# Real-time intestinal perfusion assessment for anastomotic site selection using laser speckle contrast imaging: Verification in a porcine model

**DOI:** 10.1016/j.sopen.2025.04.007

**Published:** 2025-04-18

**Authors:** Danique J.I. Heuvelings, Mahdi Al-Taher, Joost Calon, Manish Chand, Laurents P.S. Stassen, Tim Lubbers, Kevin P. Wevers, Luigi Boni, Nicole D. Bouvy, Wido Heeman

**Affiliations:** a NUTRIM School of Nutrition and Translational Research in Metabolism, Maastricht University, Maastricht, the Netherlands; b Department of Surgery, Maastricht University Medical Center, Maastricht, the Netherlands; c IRCAD, Research Institute against Digestive Cancer, Strasbourg, France; d Department of Surgery, Tawam Hospital Al-Ain, United Arab Emirates; e Wellcome EPSRC Centre for Interventional and Surgical Sciences (WEISS), University College London, London, United Kingdom; f GROW School for Oncology and Developmental Biology, Maastricht University, Maastricht, the Netherlands; g Department of General and Minimally Invasive Surgery, Fondazione IRCCS Cà Granda Ospedale Maggiore Policlinico di Milano, Milano, Italy; h Department of Surgery, University Medical Centre Groningen, Groningen, the Netherlands; i LIMIS Development BV, Leeuwarden, the Netherlands

**Keywords:** Anastomotic leakage, Image-guided surgery, Laparoscopic surgery, Laser speckle contrast imaging, Perfusion assessment

## Abstract

**Introduction:**

Adequate blood perfusion is widely recognized as a crucial factor for successful healing of an anastomosis and avoid anastomotic leakage. This study aimed to determine if laparoscopic laser speckle contrast imaging, can provide valuable feedback for identifying the state of tissue perfusion. Therefore, we explored the efficacy and feasibility of a new laser speckle contrast imaging system to assess real-time intestinal perfusion.

**Methods:**

Three gradually perfused porcine small bowel loops were created, and five senior surgeons were asked to assess the perfusion differences based on laser speckle contrast images using PerfusiX-Imaging®. Subsequently, the study evaluated the impact of laser speckle contrast imaging on decision-making for anastomosis creation. Afterwards, a questionnaire was completed by all surgeons to assess the usability of the device.

**Results:**

Results demonstrated a high accuracy (100 %) in identifying compromised perfusion and detecting perfusion differences between loops using the imaging system. In case of compromised perfusion, all surgeons recommended against creating an anastomosis based on the visual feedback. The questionnaire revealed that the senior surgeons were satisfied with the perfusion imager, particularly in terms of minimal latency, ease of use and set up, and ability to accurately represent blood flow patterns as these questions showed a (very) strong agreement in 80 %.

**Conclusion:**

Laser speckle contrast imaging can provide valuable real-time feedback on intestinal tissue perfusion during surgery, enabling surgeons to select optimal tissue segments for a well-perfused anastomosis. However, further research is required to validate the efficacy in clinical settings and its potential impact on surgical outcomes in patients.

## Introduction

Anastomotic leakage (AL) is a major complication following gastrointestinal surgery and remains the foremost concern for gastrointestinal surgeons. The occurrence rate of AL varies between 1 and 19 % depending on the anatomic location of the anastomosis [1], [2], [3], [4]. The AL etiology is influenced by various factors, including patient characteristics, peroperative factors, and tissue perfusion [2], [5], [6], [7]. Adequate blood perfusion is widely recognized as a crucial factor for successful healing of an anastomosis [1], [8], [9], [10]. Insufficient perfusion can impair the natural healing of the body, compromising the repair process and increasing the risk of AL. In recent years, there has been growing interest in utilizing real-time perfusion assessment techniques to guide surgical decision-making to improve outcomes [11]. By identifying tissue areas with compromised perfusion, surgeons can potentially avoid creating an anastomosis in those regions and opt for better-perfused tissue, thereby minimizing the risk of AL [12].

Real-time identification of intestinal perfusion to guide surgeons towards creating a bowel anastomosis using tissue with optimal perfusion, can be achieved using laser speckle contrast imaging (LSCI) [13], [14], [15] with instantaneous and continuous 2D-perfusion maps [16], [17], [18]. This imaging technique allows visualization of tissue perfusion in real-time, without the need for contrast agents [19]. By integrating the LSCI system into current laparoscopic video systems and surgical workflow, surgeons can have immediate access to visual information on tissue perfusion during the procedure. This additional feedback may serve as a valuable tool to identify regions with compromised perfusion, prompting surgeons to select alternative tissue segments for anastomoses that exhibit better perfusion [20]. If real-time identification of intestinal perfusion proves feasible and effective, it could serve as a valuable adjunct in surgical practice, providing surgeons with additional information used in better substantiated clinical decision making and optimize patient outcomes.

We hypothesized that the use of LSCI will enable surgeons to make better informed decisions regarding anastomotic site selection prompting surgeons to select alternative tissue segments for anastomosis that exhibit better perfusion, and therefore potentially reduce AL rates in future patients. Therefore, the current study aimed to assess real-time identification of intestinal perfusion using laparoscopic LSCI, subsequent decision making based on this assessment, and the efficacy and feasibility of the used LSCI device.

## Materials and methods

This study was performed at the animal facility of Maastricht University Medical Center, Maastricht, The Netherlands. The animal was treated in compliance with the regulations of the Dutch legislation concerning animal research and ARRIVE guidelines, and a protocol approved by the Local Experimental Animal Committee (DEC) (number 2017-021-001).

### Animal

A female Dutch Landrace pig weighting approximately 35 kg was used in this study. The pig underwent an acclimatization period of one week in the animal keeping facility prior to the experiment. During this period, the pig had free access to water but was fasted for 24 h. For anesthesia induction, a combination of medications was administered intravenously. This included sufentanyl at a dosage of 0.01 mg/kg/h (Hameln Pharma GmbH, Hameln, Germany), Propofol at a dosage of 9 mg/kg/h (B. Braun Melsungen AG, Melsungen, Germany), and Midazolam at a dosage of 1 mg/kg/h (Aurobindo, Baarn, The Netherlands). These medications were used to induce a state of anesthesia in the pig. The pig was mechanically ventilated to ensure adequate respiration throughout the procedure. The ventilation was adjusted as needed to maintain appropriate oxygenation and ventilation. During the procedure, anesthesia was maintained using a continuous infusion of sufentanyl and propofol and additional doses were given whenever necessary. At the end of the experimental procedure, the pig was euthanized using a lethal dose of 200 mg/kg Pentobarbital (AST Farma, Oudewater, The Netherlands).

### Laser speckle contrast imaging

**Fig. 1:**
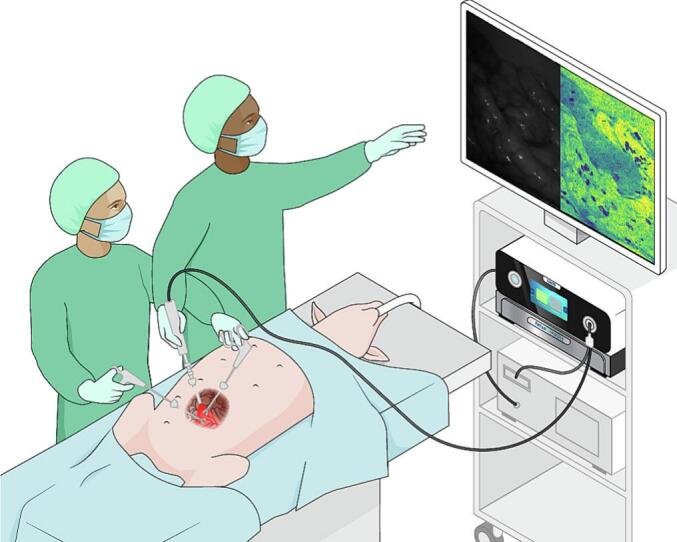
Graphic representation of the experimental setup. Illustration made by Sieben Medical Art, © 2023 Sieben Medical Art.

The PerfusiX-Imaging® device, developed by LIMIS Development BV (Leeuwarden, The Netherlands), was used for acquiring LSCI images. The system is designed to work in conjunction with standard laparoscopic equipment (Fig. 1). For this study an Olympus laparoscopic video system (OTV—S190, Olympus, Hamburg, Germany) and a 30-degree laparoscope (EndoEye, Olympus, Hamburg, Germany) were used. LSCI is a non-invasive imaging technique that offers high spatial and temporal resolution for subsurface perfusion measurements [21]. It can capture large surface areas without the need of a contrast agent. The technique leverages the coherent properties of laser light to provide real-time perfusion information. LSCI is a real-time 2D-perfusion imaging technique that relies on low power laser light to illuminate the tissue of interest. The laser light produces a random interference pattern, known as the speckle pattern, on the camera sensor. This pattern undergoes changes when underlying red blood cells move, corresponding to the rate of blood flow. Consequently, the blurring of the image or loss in contrast within the speckle pattern represents blood flow. Notably, the laparoscope was used without modification allowing the device to integrate into surgical practice. The device houses a red laser and allows for a fast, instant switching between conventional white light and laser light. As a result, 2D perfusion maps were generated in real-time and made directly available in the operating room. These maps provided visual representations of tissue perfusion, enabling immediate perfusion assessment and analysis during the surgical procedure.

### Surgical procedure and identification of three differently perfused intestinal loops

After proper sedation and analgesia, laparoscopic instruments were introduced by an experienced colorectal surgeon (M.A-T.). During the experiment, a small bowel ischemic loop model was used that was previously described by Diana et al. [22]. In short, small bowel loops with a length of approximately 15 cm were selected and arteries at the mesenteric side of the small bowel loop were transected to impair perfusion. To evaluate the ability of surgeons to identify and differentiate ischemic intestinal loops with varying levels of perfusion using both LSCI derived visual feedback and conventional white light images, three differently perfused ischemic intestinal loops were created, each measuring approximately 15 cm in length. The first loop underwent tissue perfusion compromise through the dissection of 15 arteries 90 min prior to surgeon evaluation. The second loop underwent a lesser perfusion alteration with fewer dissected arteries, with the dissection of eight arteries occurring just five minutes before questioning. The third loop had unaltered perfusion. To assess the state of perfusion, three sections of the small bowel loops were selected: the middle section of the first loop (with compromised perfusion), the end section of the second loop (with more recent perfusion alteration and less compromised perfusion), and a section from a healthy loop (with normal perfusion). The state of perfusion was confirmed by three specialists who examined white light images of the tissue. Discoloration of the tissue, previously shown to be indicative of ischemic intestinal tissue using LSCI, was considered the gold standard [18]. To mitigate potential bias from the white light images, the LSCI perfusion mode was activated when the surgeons entered the operating theatre and the (dissected) mesentery of the bowel loops was covered by a gauze for blinding. All five senior surgeons participating in the study entered the operating room individually and were asked to answer three specific questions based on the LSCI derived visual feedback. The questions posed to the surgeons were as follows: (1) Could you identify an ischemic intestinal loop? (2) Could you detect a perfusion difference in the other two loops? (3) Could you identify the best perfused loop? After responding to the questions, the surgeons were shown the corresponding white light images for further evaluation and comparison.

### Identification of anastomotic perfusion

To evaluate the ability of senior surgeons to make decisions regarding anastomosis creation based on additional visual feedback, a hand-sewn anastomosis was created using a healthy and an ischemic small bowel loop. The ischemic loop was created 30 min prior to questioning by dissecting eight peripheral arteries and veins. The state of perfusion was confirmed by three specialists based on white light images. All five senior surgeons participating in the study entered the operating room individually and were asked three specific questions based on the perfusion images. The questions posed to the surgeons were as follows: (1) Would you advise creating an anastomosis based on this additional visual feedback? (2) Can you identify a perfusion difference? and (3) What is the worst perfused tissue?

### Usability of PerfusiX-imaging for intestinal perfusion assessment

A questionnaire was designed to assess the usability of the device. The questionnaire consisted of six items, each addressing a specific aspect of usability. The items were answered using the Likert scale from one to five, with the one representing the least favorable response and five indicating the most favorable response. The questionnaire can be found in Supplemental material S1.

## Results

### Animal experiment and surgical procedure

The surgical procedure was performed without any complications nor adverse events.

### Identification of three differently perfused intestinal loops

**Fig. 2:**
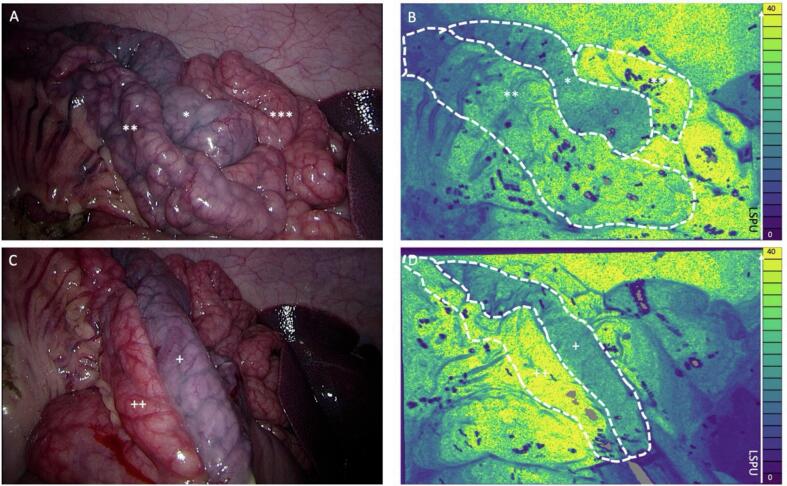
**(A)** The white light image of the three differently perfused loops. * Indicates the ischemic loop, ** indicates the compromised loop and *** indicates the healthy loop. **(B)** The PerfusiX-Imaging® perfusion image with the three differently perfused loops. Blue indicates low perfusion and yellow indicates high perfusion. * Indicates the ischemic loop, ** indicates the compromised loop and *** indicates the healthy loop. **(C)** The white light image of the anastomosis with a bad perfused segment indicated by + and an uncompromised segment indicated with ++. **(D)** The PerfusiX-Imaging perfusion image of the anastomosis. Blue indicates low perfusion and yellow indicates high perfusion. The compromised segment indicated by + and an uncompromised segment indicated with ++. (For interpretation of the references to colour in this figure legend, the reader is referred to the web version of this article.)

The results indicated that surgeons demonstrated a good ability to identify ischemic intestinal loops using LSCI derived visual feedback (Fig. 2). Specifically, all five senior surgeons correctly identified the ischemic loop, achieving a 100 % accuracy rate when relying solely on this feedback. After the identification of the ischemic bowel loop using only LSCI, the white light images were shown. All surgeons still agreed with the identified ischemic region and no one doubted his/hers decision based on this additional information. Regarding the ability to detect perfusion differences in the other two loops, again LSCI derived visual feedback enabled all five surgeons to accurately identify the differences, resulting in a 100 % accuracy rate. When asked to identify the best perfused loop, four out of five (80 %) surgeons provided correct answers based solely on the laser speckle images.

### Identification of anastomotic perfusion

The study's findings demonstrated that the LSCI perfusion images had an impact on the surgeons' decision-making concerning anastomosis creation. All five senior surgeons, when presented with LSCI feedback (Fig. 2B and D), recommended against creating an anastomosis, resulting in a recommendation rate of 100 %. In terms of identifying perfusion differences, the LSCI feedback alone proved to be highly effective, as all surgeons correctly identified the differences, leading to a 100 % accuracy rate. Similarly, when asked to identify the worst perfused tissue, all surgeons provided correct answers based solely on the LSCI feedback. The inclusion of white light images did not alter the accuracy in this regard.

### Usability for intestinal perfusion assessment

**Fig. 3:**
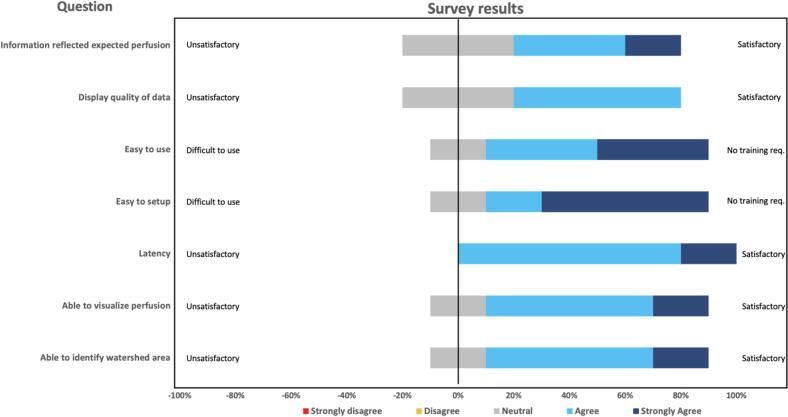
The results from the survey filled in by five surgeons represented in percentages in a Likert profile graph. The answers ranged from strongly disagree/bad to strongly agree/good. The questions were (1) The PerfusiX-Imaging perfusion information reflected the expected pattern of blood flow? (2) How was the display quality of PerfusiX-Imaging on displaying the blood flow? (3) PerfusiX-Imaging was easy to use during surgery? (4) How easy was it to setup PerfusiX-Imaging? (5) Was there latency during the perfusion imaging? (6) PerfusiX-Imaging was able to visualize tissue perfusion intraoperatively? And (7) I was able to identify the intestinal watershed area using PerfusiX-Imaging.

The collected data from the questionnaire were analyzed to assess the usability of PerfusiX-Imaging®. The questionnaire was filled in by five senior surgeons. No one disagreed on any of the questions. All surgeons (strongly) agreed on the minimal latency during the surgical procedure. Besides, 80 % of the surgeons (*n* = 4) (strongly) agreed that the system was easy to use, easy to set up, able to visualize perfusion and able to visualize watershed areas. Two surgeons agreed and one strongly agreed (total of 60 %) on the statement that the LSCI information reflected the expected pattern of blood flow. An additional 60 % agreed on the good quality of the displayed data. The results from the survey are displayed in percentages in Fig. 3.

## Discussion

In the current animal study, we successfully acquired laser speckle contrast images during intestinal surgery using laparoscopic LSCI setup, demonstrated the capability of indicating ischemic bowel regions with this technique, and demonstrated the usability of LSCI system for intestinal perfusion assessment.

The use of LSCI feedback allowed us to visualize and detect differences of intestinal perfusion, which can serve as a critical indicator of tissue perfusion. The noticeable attributes of LSCI appear especially captivating when applied to the creation of intestinal anastomoses. In this context, it becomes imperative to conduct an intraoperative evaluation of intestinal microperfusion to confirm the vitality of the recently established anastomosis, aiming to avert complications stemming from insufficient blood supply, such as AL. The conventional approach of the surgeon relying on visual examination has demonstrated marked subjectivity and offers minimal predictive efficacy [12]. The latter stimulated the advancement of perfusion imaging methods, especially near-infrared fluorescence imaging [22], [23]. Fluorescence angiography has some distinct disadvantages compared to LSCI. These include the need for a fluorescence dye and the inability to repetitively and continuously assess bowel perfusion due to wash-out effects [24], [25]. In contrast, we were able to detect the location of the intestinal watershed area in real time without the need to administer an exogenous dye with LSCI. Identifying the location of the intestinal watershed area with LSCI could serve as a so-called red flag technique in guiding surgeons towards an anastomosis created with better perfused tissue. The ability to assess anastomotic perfusion in real-time provides surgeons with important information that complements their conventional assessment methods. This additional feedback empowers surgeons to detect perfusion differences between tissue segments and identify the worst and best perfused tissue more accurately [16], [17], [18], [20].

LSCI is still seen as a less commonly used perfusion imaging technique, although its clinical applications seem promising. Previous animal studies already published promising results in its ability to distinguish between well-perfused and poor-perfused bowel tissue [26], [27] as well as in assessing renal perfusion [28], [29] and the perfusion of the gastric conduit [30]. In contrast to other imaging techniques such as near-infrared fluorescence imaging (NIRF), there is no need for any pharmaceuticals or dyes. Additionally, LSCI captures the possibility for continued perfusion evaluation, without difficulties such as residual signals or wash-out effects [31]. Future validation and exploration are necessary to assess the exact value of this current red flag technique in colorectal surgery. In the current study the surgeons were asked to draw conclusions based on LSCI images (verification). Although, for clinical setting it may be interesting to perform the current study in opposite direction and investigate if LSCI provides additional information compared to the white light images (validation). Additionally, it is interesting to compare these outcomes with other imaging technique such as fluorescence angiography as previous research has already shown the complemental role of LSCI compared to NIRF in parenchymal perfusion assessment [32]. Yet, it may also be interesting to deeper evaluate inter-observer variability of experienced surgeons as used in this study, compared to less experienced residents. LSCI could emerge as a more direct, real-time, and repeatable approach in providing quantitative information on tissue perfusion [32], [33], [34]. Developing additional methods to quantify the LSCI output can extra enhance the accuracy and reliability of LSCI. Given that this research exclusively focusses on establishing the practicability of measuring perfusion and identifying perfusion discrepancies at the anastomosis, future studies should prioritize evaluating the device's performance in clinical trials and examining surgical outcomes, such as anastomotic leakage (AL) rates and overall patient recovery. Ideally, these studies should also focus on validating quantification methods for LSCI to facilitate easier comparisons of both clinical and research outcomes. Furthermore, research should explore the device's potential application in both laparoscopic and robotic procedures.

The results from the survey indicated that the senior surgeons were overall very satisfied with PerfusiX-Imaging® as a perfusion imager and its use during the surgical procedure. The system's ability to accurately represent blood flow patterns, high display quality of data, ease of use, efficient setup, minimal latency, and real-time visualization of tissue perfusion were positively acknowledged. However, it is important to note that this study was conducted in an animal model, and further research is needed to validate the efficacy and feasibility of the LSCI device in clinical settings. Besides, while anastomotic perfusion is commonly required during colorectal anastomotic creation, we used small bowel loops in our experiment. This decision was based on the difficult curly nature of a pig's colon, making the small bowel a more suitable choice for illustrative and surgical technical purposes. Consequently, the generalizability of our findings to human patients should be further investigated. Yet, previous research has shown good results using the same device for colonic perfusion assessment in a human population [20]. Also, for this experiment we conducted numerous experiments with separate bowel loops, however, it is essential to note that these observations were derived from a single animal. Consequently, it is imperative to be cautious when interpreting the data presented in this study, given its limited sample size. For our animal studies, we consider it of paramount importance to adhere to the principles of the 3R's: replacement, reduction, and refinement [35]. As such, the current experimental design was considered adequate for assessing the hypothesis.

## Conclusion

The PerfusiX-Imaging® device provided visual feedback for assessing reduced intestine perfusion in ischemic loops, detecting perfusion differences between loops, and identifying the best perfused loop in a porcine model. The real-time 2D-perfusion maps offered immediate and continuous information on tissue perfusion, which may help to select optimal sites for anastomosis creation. Although surgeons were overall very satisfied with using the system, further research is required to validate the efficacy in clinical settings and its potential impact on surgical outcomes in patients.

The following is the supplementary data related to this article.

Supplementary material S1 Usability of PerfusiX-Imaging for intestinal perfusion assessment questionnaire. Supplementary material S1

## CRediT authorship contribution statement

**Danique J.I. Heuvelings:** Writing – review & editing, Writing – original draft, Visualization, Project administration, Methodology, Investigation, Formal analysis, Data curation, Conceptualization. **Mahdi Al-Taher:** Writing – review & editing, Methodology, Conceptualization. **Joost Calon:** Writing – review & editing. **Manish Chand:** Writing – review & editing. **Laurents P.S. Stassen:** Writing – review & editing. **Tim Lubbers:** Writing – review & editing, Methodology, Investigation. **Kevin P. Wevers:** Writing – review & editing, Writing – original draft, Methodology, Conceptualization. **Luigi Boni:** Writing – review & editing. **Nicole D. Bouvy:** Writing – review & editing, Writing – original draft, Methodology. **Wido Heeman:** Writing – review & editing, Writing – original draft, Methodology, Investigation, Formal analysis, Data curation, Conceptualization.

## Contributions

All authors have made substantial contributions to the conception and design of the study, or acquisition of data, or analysis and interpretation of data, drafting the article or revising it critically for important intellectual content, and final approval of the version to be submitted.

## Ethical

The animal was treated in compliance with the regulations of the Dutch legislation concerning animal research and a protocol approved by the Local Experimental Animal Committee (DEC) (number 2017-021-001).

## Funding

Not applicable.

## Declaration of competing interest

Laurents P.S. Stassen is a member of the advisory board of Diagnostic Green GmbH. Nicole D. Bouvy is a member of the advisory board of Active Surgical and received an education grant from Medtronic. Manish Chand received consulting fees from Arthrex and Intuitive Surgical imaging, and stock options for Activ Surgical. Luigi Boni received consulting fees from Storz, Arthrex, Olympus, CMR, and Intutive. Wido Heeman and Joost Calon are employees of LIMIS Development and ZiuZ Visual Intelligence respectively. Danique J.I. Heuvelings, Mahdi Al-Taher, Tim Lubbers and Kevin Wevers have no conflicts of interest nor financial ties to disclose.

## Data Availability

Data supporting this study are included within the manuscript and supporting materials. More information can be gained through contacting the corresponding author.
