# Supplementary material for: Real-time intestinal perfusion assessment for anastomotic site selection using laser speckle contrast imaging: Verification in a porcine model
Source: Surg Open Sci. 2025 Apr 18;26:12–7. doi: 10.1016/j.sopen.2025.04.007 (PMC12417363; doi:10.1016/j.sopen.2025.04.007)
Supplement: Supplementary material S1 — Usability of PerfusiX-Imaging for intestinal perfusion assessment questionnaire. [file mmc1.docx]

## **S1. Usability of PerfusiX-Imaging for intestinal perfusion assessment questionnaire**

A questionnaire was designed to assess the usability of PerfusiX-Imaging. The questionnaire consisted of six items, each addressing a specific aspect of usability. The items were answered using the Likert scale from 1 to 5, with the 1 representing the least favorable response and 5 indicating the most favorable response. The questionnaire can be found underneath.

| 1. | I was able to identify the intestinal watershed area using PerfusiX-Imaging |
| --- | --- |
| 2. | PerfusiX-Imaging was able to visualize tissue perfusion intraoperatively |
| 3. | Was there latency during the perfusion imaging? |
| 4. | How easy was it to setup PerfusiX-Imaging? |
| 5. | PerfusiX-Imaging was easy to use during surgery |
| 6. | How was the display quality of PerfusiX-Imaging on displaying the blood flow? |
| 7. | The PerfusiX-Imaging perfusion information reflected the expected pattern of blood flow |
